# Pathophysiological Association between Diabetes Mellitus and Endothelial Dysfunction

**DOI:** 10.3390/antiox10081306

**Published:** 2021-08-18

**Authors:** Tatsuya Maruhashi, Yukihito Higashi

**Affiliations:** 1 Department of Cardiovascular Regeneration and Medicine, Research Institute for Radiation Biology and Medicine, Hiroshima University, Hiroshima 734-8553, Japan; maru0512@hiroshima-u.ac.jp; 2 Division of Regeneration and Medicine, Medical Center for Translational and Clinical Research, Hiroshima University Hospital, Hiroshima 734-8551, Japan

**Keywords:** diabetes mellitus, endothelial dysfunction, oxidative stress

## Abstract

Endothelial dysfunction plays a critical role in atherosclerosis progression, leading to cardiovascular complications. There are significant associations between diabetes mellitus, oxidative stress, and endothelial dysfunction. Oxidative stress is increased by chronic hyperglycemia and acute glucose fluctuations induced by postprandial hyperglycemia in patients with diabetes mellitus. In addition, selective insulin resistance in the phosphoinositide 3-kinase/Akt/endothelial nitric oxide (NO) synthase pathway in endothelial cells is involved in decreased NO production and increased endothelin-1 production from the endothelium, resulting in endothelial dysfunction. In a clinical setting, selecting an appropriate therapeutic intervention that improves or augments endothelial function is important for preventing diabetic vascular complications. Hypoglycemic drugs that reduce glucose fluctuations by decreasing the postprandial rise in blood glucose levels, such as glinides, α-glucosidase inhibitors and dipeptidyl peptidase 4 inhibitors, and hypoglycemic drugs that ameliorate insulin sensitivity, such as thiazolidinediones and metformin, are expected to improve or augment endothelial function in patients with diabetes. Glucagon-like peptide 1 receptor agonists, metformin, and sodium-glucose cotransporter 2 inhibitors may improve endothelial function through multiple mechanisms, some of which are independent of glucose control or insulin signaling. Oral administration of antioxidants is not recommended in patients with diabetes due to the lack of evidence for the efficacy against diabetic complications.

## 1. Introduction

The number of patients with diabetes has been increasing worldwide. A pooled analysis showed that the prevalence of diabetes in adults has been increasing, and it was estimated that the number of adults with diabetes in the world increased from 108 million in 1980 to 422 million in 2014 [1]. Diabetic patients are at high risk for developing microvascular and macrovascular complications. Diabetic vascular complications are major causes of morbidity and mortality in diabetic patients. Microvascular complications such as neuropathy, nephropathy, and retinopathy are major causes of decreased quality of life. Macrovascular complications including coronary heart disease, cerebrovascular disease, and peripheral artery disease, are major causes of mortality. A systematic literature review has shown that cardiovascular disease affects approximately 32.2% of patients with type 2 diabetes (21.1% with coronary heart disease, 14.6% with angina, 10.0% with myocardial infarction, and 7.6% with stroke) and that cardiovascular disease is the cause of death in 9.9% of patients with type 2 diabetes aged 63.6 ± 6.9 years, accounting for 50.3% of all deaths in diabetic patients [2]. Therefore, inhibiting the progression of atherosclerosis and preventing the development of diabetic vascular complications is clinically important for a better prognosis in diabetic patients.

Endothelial dysfunction is not only an early event of atherosclerosis but also plays a critical role in atherosclerosis progression, leading to the development of vascular complications [3,4,5]. Diabetes mellitus is associated with endothelial dysfunction [6,7,8,9]. Therefore, it is important to understand the mechanisms underlying endothelial dysfunction caused by diabetes mellitus and to select treatments that improve or augment endothelial function for preventing diabetic vascular complications. In this review, we discuss the current understanding of the mechanisms of endothelial dysfunction and recommended therapeutic options for improving endothelial function in patients with diabetes mellitus.

## 2. The Resting Endothelium

The vascular endothelium acts as an endocrine organ that secrets a variety of vasoactive agents: vasodilators such as prostaglandin I_2_, endothelium-derived hyperpolarizing factor, and nitric oxide (NO), and vasoconstrictors such as angiotensin II, thromboxane A_2_, and endothelin-1 (ET-1) [10]. The resting endothelium acts as a gatekeeper that maintains vascular homeostasis by regulating the moment-to-moment balance between vasodilation and vasoconstriction, antithrombosis and prothrombosis, anti-inflammation and proinflammation, antioxidation and pro-oxidation, and vascular smooth muscle cell growth inhibition and growth promotion [3]. Endothelial dysfunction refers to the inability of endothelial cells to maintain vascular homeostasis due to the disturbed balance between endothelium-derived proatherosclerotic factors and antiatherosclerotic factors in favor of proatherosclerotic factors, leading to the initiation and progression of atherosclerosis. NO released from the endothelium has a variety of antiatherosclerotic effects such as vasodilation, inhibition of the proliferation of vascular smooth muscle cells, inhibition of leukocyte adhesion, and inhibition of platelet adhesion and aggregation. Therefore, endothelial dysfunction often refers to a condition in which increased NO inactivation and/or decreased NO production from the endothelium results in reduced NO bioavailability.

Diabetes mellitus is associated with endothelial dysfunction [11,12,13]. Clinical studies have shown that endothelial function assessed by endothelium-dependent vasodilation is impaired in diabetic patients [14,15]. Although the pathogenesis has not been fully elucidated, several mechanisms underlying the relationship between diabetes mellitus and endothelial dysfunction have been proposed. 

## 3. Mechanisms Underlying Endothelial Dysfunction in Diabetes Mellitus

### 3.1. Oxidative Stress

Reactive oxygen species (ROS) are derived from molecular oxygen. Oxidative stress refers to the disturbed balance between the antioxidant system and ROS in favor of ROS. When the counteracting effect of the antioxidant system is insufficient, harmful effects of ROS such as inhibition of signal transduction pathways or normal cellular functions through damage to cellular lipids, proteins, or DNA become evident. In human cells, ROS are produced by various enzymatic sources such as nicotinamide-adenine dinucleotide phosphate (NADPH) oxidases, xanthine dehydrogenase/oxidase, the mitochondrial electron transport chain, uncoupled endothelial NO synthase (eNOS), cyclooxygenase, lipoxygenase, and glucose oxidase [16]. ROS include free radical species such as superoxide anion radical (O_2_^•−^), peroxyl radical, alkoxyl radical, and hydroxyl radical, and non-radical species such as singlet molecular oxygen, hydrogen peroxide, organic hydroperoxides, hypochlorous acid, and ozone [16].

There is an interaction between endothelial function and oxidative stress [3,17]. O_2_^•−^, one of the free radical species, is produced through the removal of one electron from molecular oxygen. NO is directly inactivated by O_2_^•−^ with high affinity, resulting in decreased NO bioavailability. In addition, peroxynitrite is produced as a result of the direct reaction between NO and O_2_^•−^ [18]. Peroxynitrite is a highly potent oxidant that can cause lipid peroxidation, protein tyrosine nitration, DNA damage, and cell death [18]. Tetrahydrobiopterin (BH_4_), an essential eNOS cofactor, is oxidized by peroxynitrite to the biologically inactive form, resulting in reduced BH_4_ availability. Under a condition of insufficient BH_4_, O_2_^•−^ is produced instead of NO from uncoupled eNOS [19]. Therefore, O_2_^•−^ is closely associated with the development of endothelial dysfunction. Once an oxidative condition is established, endothelial function continues to be impaired through a vicious cycle of increased O_2_^•−^ and decreased NO bioavailability. In diabetes mellitus, chronic hyperglycemia, acute glucose fluctuations, and insulin resistance are regarded as major causes of endothelial dysfunction.

### 3.2. Chronic Hyperglycemia and Oxidative Stress

**Figure 1:**
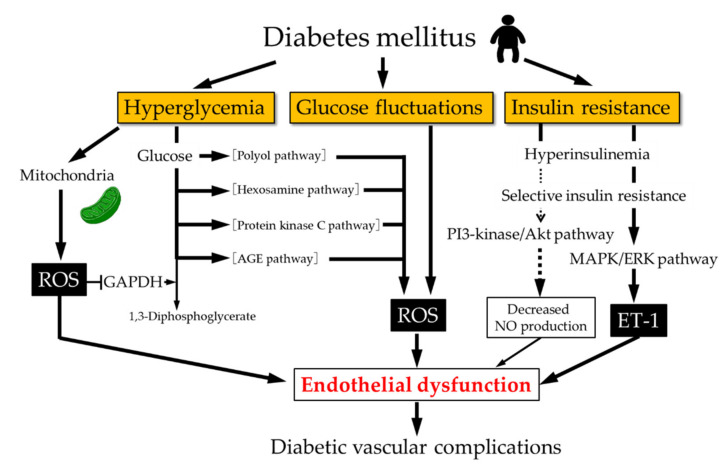
Mechanisms of endothelial dysfunction in diabetic patients. Chronic hyperglycemia, acute glucose fluctuations, and insulin resistance are involved in endothelial dysfunction in diabetic patients. ET-1, endothelin-1; ROS, reactive oxygen species; AGE, advanced glycation end products; PI3-kinase, phosphoinositide 3-kinase; MAPK, mitogen-activated protein kinase; ERK, extracellular signal-regulated kinase.

Intracellular O_2_^•−^ is mainly produced from mitochondria [20]. Pyruvate is generated by glycolysis in the cytosol and is used for ATP synthesis by oxidative phosphorylation in mitochondria. After the transportation of pyruvate into the mitochondria, pyruvate is oxidized by the tricarboxylic acid (TCA) cycle to produce H_2_O, CO_2_, nicotinamide adenine dinucleotide (NADH), and 1,5-dihydro-flavin adenine dinucleotide (FADH_2_) [21]. Electrons from mitochondrial NADH and FADH_2_ are used as energy for ATP synthesis by the electron-transport chain at the inner mitochondrial membrane. Electrons from NADH and FADH_2_ are transferred through the mitochondrial electron-transport chain, which is coupled with proton pumping by the electron-transport chain from the mitochondrial matrix into the intermembrane space. As a result of proton pumping, a proton gradient is generated across the inner membrane of mitochondria, which provides the energy for driving the ATP synthase. In a hyperglycemic state, increased production of NADH and FADH_2_ by the TCA cycle leads to increased transportation of NADH and FADH_2_ to the electron-transport chain. Since NADH and FADH_2_ serve as electron donors, electron transfer and proton pumping through the electron-transport chain is concomitantly enhanced and a proton gradient across the inner mitochondrial membrane is increased. As a result, electron transfer and proton pumping are inhibited, resulting in an increase in electron leak from the electron-transport chain and a subsequent increase in O_2_^•−^ generation in mitochondria (Figure 1) [21].

GAPDH is the glycolytic enzyme that is essential for maintaining glycolysis. Hyperglycemia-induced overproduction of mitochondrial O_2_^•−^ partially inhibits the activity of GAPDH. Therefore, the inhibition of GAPDH activity by mitochondrial O_2_^•−^ causes the accumulation of glycolytic metabolites upstream of GAPDH and the increased flux of upstream metabolites into pathways of glucose overutilization (Figure 1) [21].

An increase in glucose flux into the polyol pathway leads to increased consumption of NADPH. NADPH is required for the regeneration of reduced glutathione. Therefore, intracellular concentrations of reduced glutathione are decreased as a result of increased consumption of NADPH by an increase in glucose flux into the polyol pathway. Since reduced glutathione is a main intracellular antioxidant, intracellular oxidative stress is enhanced, leading to endothelial dysfunction.

Increased glucose flux into the hexosamine pathway may result in endothelial dysfunction. Fructose-6-phosphate is converted to glucosamine-6-phosphate in the hexosamine pathway, resulting in an increase in UDP-N-acetylglucosamine, which is required for reactions such as proteoglycan synthesis and *O*-linked glycoproteins formation. As a result of increased UDP-N-acetylglucosamine, transcriptional factors, nuclear proteins, and cytoplasmic proteins are modified by *O*-linked N-acetylglucosamine, leading to many alterations in both gene and protein functions. For instance, eNOS activity is inhibited by *O*-acetylglucosaminylation of the eNOS protein at the Akt site [22], leading to decreased NO production and consequent endothelial dysfunction.

Hyperglycemia-induced activation of protein kinase C (PKC) through an increase in diacylglycerol has a number of pathogenic effects such as decreased eNOS expression, increased ET-1 expression, increased plasminogen activator inhibitor-1 expression, increased transforming growth factor-β expression, NF-kB activation, and NADPH oxidase activation, leading to endothelial dysfunction.

Increased intracellular production of advanced glycation end products (AGE) precursors causes modifications of plasma proteins and extracellular matrix proteins and functional alterations of intracellular proteins. ROS production and NF-kB activation are induced by the activation of the receptor for AGEs on the surface of endothelial cells, leading to endothelial dysfunction. Therefore, endothelial dysfunction is caused by hyperglycemia-induced overproduction of mitochondrial O_2_^•−^ and diversion of glycolytic flux from the normal glycolytic pathway to alternative metabolic pathways due to inhibition of GAPDH activity by mitochondrial O_2_^•−^.

### 3.3. Acute Glucose Fluctuations and Oxidative Stress

In healthy subjects, plasma glucose levels are controlled and maintained within a narrow range, whereas blood glucose levels are rapidly and greatly increased in the postprandial phase in patients with diabetes mellitus. Experimental and clinical studies have indicated that endothelial function is impaired by postprandial acute hyperglycemia through increased oxidative stress in patients with diabetes mellitus (Figure 1) [23]. Compared with constant high glucose, intermittent high glucose may have more harmful effects on endothelial cells. In vitro studies have shown that more apoptosis of endothelial cells was induced by intermittent high glucose through PKC activation and NADPH oxidase activation than by constant high glucose [24,25]. In addition, clinical studies have indicated that endothelial function is impaired by glucose fluctuations through oxidative stress. Monnier et al. reported that a marker of glucose fluctuations was strongly correlated with a marker of oxidative stress, whereas there was no significant correlation between the oxidative stress marker and any other glycemic parameters such as fasting plasma glucose and glycated hemoglobin (HbA1c) [26]. Torimoto et al. reported that the marker of glucose fluctuations was negatively correlated with a marker of endothelial function [27]. These findings suggest that endothelial function is impaired by glucose fluctuations through increased oxidative stress. Although the precise molecular mechanisms underlying the association between glucose fluctuations and enhanced oxidative stress have not been fully elucidated, it is postulated that cellular metabolic adaptation to toxic effects induced by high glucose may be facilitated by a constant high glucose concentration through constant feedback. In contrast, intermittent high glucose may fail to facilitate such adaptations to toxic effects induced by high glucose because of the lack of constant feedback, leading to higher oxidative stress and consequent endothelial dysfunction. HbA1c has been used as a therapeutic marker of glucose control. HbA1c, however, reflects time-averaged glucose exposure but not glucose fluctuations. Large clinical randomized studies in which HbA1c was used as a marker of glycemic control failed to demonstrate the superiority of intensive glucose control for prevention of cardiovascular events [28,29,30]. Therefore, it is necessary to pay attention not only to HbA1c and fasting plasma glucose levels but also to postprandial glucose levels for protecting the endothelium from oxidative injury caused by postprandial hyperglycemia.

### 3.4. Selective Insulin Resistance-Induced Endothelial Dysfunction

Insulin stimulates NO production in endothelial cells. Insulin receptor substrate (IRS) is phosphorylated by the binding of insulin to its cognate receptor on endothelial cells, resulting in the activation of the phosphoinositide 3-kinase (PI3-kinase)/Akt pathway. eNOS is phosphorylated at Ser^1177^ by Akt, resulting in increased NO production [31,32]. Insulin also stimulates ET-1 production in endothelial cells through activating the mitogen-activated protein kinase (MAPK)/extracellular signal-regulated kinase (ERK) pathway independent of the PI3-kinase/Akt/eNOS pathway [33]. The PI3-kinase/Akt/eNOS pathway is selectively impaired under the condition of insulin resistance because of reduced IRS expression in endothelial cells. In contrast, the MAPK/ERK/ET-1 pathway remains unchanged and is preferentially stimulated because of the compensatory hyperinsulinemia, leading to endothelial dysfunction (Figure 1) [34]. This phenomenon is referred to as selective insulin resistance.

### 3.5. Endothelial Dysfunction in Type 1 Diabetes

Endothelial function has been shown to be impaired in patients with type 1 diabetes [15,35]. Oxidative stress is increased and antioxidant defense is impaired in patients with type 1 diabetes [36,37,38], suggesting that oxidative stress is involved in endothelial dysfunction in patients with type 1 diabetes. Clinical studies have indicated that chronic hyperglycemia and acute hyperglycemia are associated with endothelial dysfunction in patients with type 1 diabetes [39,40,41]. In addition, experimental studies have indicated the possibility that dysregulated autoimmune response in type 1 diabetes may contribute to endothelial dysfunction by increasing oxidative stress through activation of NADPH oxidase [42]. It remains unclear whether glucose fluctuations are involved in endothelial dysfunction in patients with type 1 diabetes due to the paucity of clinical evidence.

## 4. Recommended Pharmacotherapies from a Perspective of Endothelial Dysfunction in Diabetes Mellitus

Selecting an appropriate intervention that will effectively improve or augment endothelial function is clinically important for preventing cardiovascular events in diabetic patients. Considering that endothelial function may be impaired by chronic hyperglycemia, acute glycemic variability, and insulin resistance through increased oxidative stress, behavior modification and pharmacotherapies aimed at reducing blood glucose levels without hypoglycemia, reducing glucose variability, and ameliorating insulin sensitivity are expected to improve endothelial function.

### 4.1. Insulin Treatment

Intensive glucose control with insulin has been shown to reduce microvascular and macrovascular complications in patients with type 1 diabetes [43]. Insulin therapy may be beneficial for endothelial function in patients with type 1 diabetes who have a healthy energy balance without insulin resistance since there is little concern about selective insulin resistance. In contrast, the effect of insulin treatment on endothelial function may depend on the achieved level of metabolic control in patients with type 2 diabetes [44]. In obese or overweight patients with type 2 diabetes who have insulin resistance due to overnutrition and a positive energy balance, endothelial function is potentially impaired by high-dose insulin therapy due to selective insulin resistance.

### 4.2. Hypoglycemic Drugs

Sulfonylureas are insulin secretagogues. Therefore, sulfonylureas, as well as high-dose insulin therapy, could have an adverse effect on endothelial function in overweight or obese diabetics because of the selective insulin resistance, potentially resulting in endothelial dysfunction (Table 1).

Hypoglycemic drugs that reduce glucose fluctuations by decreasing postprandial hyperglycemia are expected to ameliorate endothelial function through decreasing oxidative stress. Glinides, α-glucosidase inhibitors, and dipeptidyl peptidase 4 (DPP-4) inhibitors are hypoglycemic drugs that decrease postprandial hyperglycemia. Indeed, clinical studies have shown that those hypoglycemic drugs improve postprandial endothelial function in diabetic patients [45,46,47,48]. However, there are conflicting reports regarding the effects of glinides, α-glucosidase inhibitors, and DPP-4 inhibitors on postprandial endothelial function; some studies have shown that glinides, DPP-4 inhibitors, and α-glucosidase inhibitors have no beneficial effects on endothelial function [45,49,50]. As for DPP-4 inhibitors and glinides, increased secretion of endogenous insulin through their pharmacological actions is possibly associated with a lack of a beneficial effect of DPP-4 inhibitors or glinides on endothelial function because of the selective insulin resistance in overweight or obese patients with diabetes mellitus.

Glucagon-like peptide 1 receptor (GLP-1R) agonists improve the control of postprandial blood glucose levels. Therefore, GLP-1R agonists are expected to improve endothelial function through reducing glucose fluctuations in patients with diabetes mellitus. Indeed, GLP-1R agonists have been shown to augment endothelial function in patients with diabetes mellitus [51,52,53,54,55]. However, augmentation of postprandial endothelial function by GLP-1R agonists was independent of postprandial changes in blood glucose levels or insulin sensitivity but was associated with postprandial reduction in serum levels of triglycerides [51,52,54]. It has also been shown that eNOS activation in endothelial cells through GLP1-R and AMP-activated protein kinase (AMPK) activation is involved in the augmentation of endothelial function by GLP-1R agonists [53].

Considering that there is a significant association between endothelial dysfunction and insulin resistance, insulin sensitizers are anticipated to ameliorate endothelial function through restoring PI3-kinase/Akt/eNOS pathway and increasing NO production. Indeed, clinical studies have shown that thiazolidinediones, which are insulin sensitizers, improve endothelium-dependent vasodilation [56,57].

Metformin, which is an insulin sensitizer, has been shown to ameliorate endothelial function with a significant association between endothelial function and insulin resistance following treatment in patients with type 2 diabetes mellitus [58]. In addition, metformin has been shown to improve endothelial function in patients without diabetes who have insulin resistance [59]. These findings suggest that metformin improves endothelial function by ameliorating insulin resistance. On the other hand, other clinical studies have shown that metformin improves endothelial dysfunction irrespective of insulin sensitivity or body weight, suggesting that metformin improves endothelial function through multiple mechanisms, some of which are independent of insulin resistance [60,61]. Preclinical studies have shown that metformin improves endothelial function by eNOS phosphorylation through AMPK activation [62,63], sirtuin-1 activation [64,65], and promotion of antioxidation [66].

Sodium-glucose cotransporter 2 (SGLT2) inhibitors lower blood glucose levels by increasing glucose excretion into urine through inhibition of renal glucose reabsorption [67]. The glucose-lowering effect of SGLT2 inhibitors is independent of insulin. Therefore, there is little concern that treatment with SGLT2 inhibitors in overweight or obese diabetics with insulin resistance would further deteriorate endothelial function through the mechanism of selective insulin resistance in endothelial cells. SGLT2 inhibitors have been shown to reduce postprandial glucose levels and decrease overall glucose variability in patients with diabetes mellitus [68,69]. In addition, SGLT2 inhibitors have been shown to reduce insulin resistance and ameliorate peripheral insulin sensitivity [70,71,72]. SGLT2 inhibitors have other metabolic actions including reductions in plasma lipid levels, blood pressure, and body weight. Therefore, SGLT2 inhibitors are expected to improve or augment endothelial function by decreasing oxidative stress through lowering glucose levels in an insulin-independent manner, reducing acute glucose fluctuations, improving insulin sensitivity, and improving other metabolic parameters. Indeed, an experimental study demonstrated that urinary excretion of 8-oxo-2′-deoxyguanosine, an oxidative stress marker, was decreased and endothelial function was ameliorated by treatment with an SGLT2 inhibitor in mice [73]. In addition, clinical studies have shown that SGLT2 inhibitors improve endothelial function in patients with diabetes mellitus [74,75,76,77,78].

### 4.3. Other Treatment

Dyslipidemia and hypertension are significantly associated with endothelial dysfunction and are highly prevalent in patients with type 2 diabetes [79,80]. Therefore, a multiple-risk-factor intervention approach should be performed to improve endothelial function and prevent future cardiovascular events. The Steno-2 Study demonstrated that the risk of microvascular complications and cardiovascular events was significantly reduced by an intensified, target-driven, multifactorial intervention involving a combination of focused behavior modification and medications aimed at concomitant cardiovascular risk factors than by a conventional strategy [81,82]. In the treatment of other modifiable risk factors in diabetic patients, it is recommended to select an intervention that ameliorates endothelial function, such as administration of statin [83], administration of blockers of the renin-angiotensin system [84,85,86], and behavior modifications such as body weight reduction, aerobic exercise, and smoking cessation [87,88,89,90].

Since oxidative stress has been considered as a major cause of endothelial dysfunction in patients with diabetes, an intervention targeting direct reduction in oxidative stress is attractive and expected to improve endothelial function and cardiovascular outcomes in patients with diabetes. Indeed, endothelial function is augmented by concomitant intra-arterial infusion of vitamin C in patients with diabetes [91,92]. However, oral administration of antioxidants, including vitamin C, vitamin E and N-acetylcysteine, has failed to demonstrate a protective effect of antioxidants against diabetic vascular complications in patients with diabetes [93,94,95,96]. Although the precise reasons for the ineffectiveness of oral administration of antioxidants remain unclear, ineffectiveness may be due to the lack of pharmacokinetic evaluation; plasma levels of the supplemented antioxidants were not monitored and the drug range for safety and efficacy was not determined [12]. Therefore, administration of antioxidants for the prevention of diabetic vascular complications is not clinically recommended in patients with diabetes.

## 5. Conclusions

Endothelial dysfunction is a therapeutic target in patients with diabetes mellitus. Oxidative stress induced by chronic hyperglycemia, acute glucose fluctuations, and decreased NO production by selective insulin resistance in endothelial cells may be associated with endothelial dysfunction in patients with diabetes. In addition to behavior modifications including body weight reduction, aerobic exercise, and smoking cessation, hypoglycemic drugs that reduce acute glucose fluctuations, such as glinides, α-glucosidase inhibitors and DPP-4 inhibitors, and hypoglycemic drugs that ameliorate insulin sensitivity, such as thiazolidinediones and metformin, are expected to improve endothelial function in patients with diabetes mellitus. Preclinical studies have indicated the possibility that GLP1-R agonists, metformin, and SGLT2 inhibitors improve endothelial function by multiple mechanisms, some of which are independent of glucose control or insulin signaling, such as eNOS phosphorylation through AMPK and sirtuin-1 activation. Selecting appropriate hypoglycemic drugs that will improve or augment endothelial function may be clinically important to prevent diabetic vascular complications for better prognosis in diabetic patients. In the treatment of other modifiable risk factors, including hypertension and dyslipidemia, in patients with diabetes, it is recommended to select an appropriate intervention that will improve endothelial function, such as administration of statins and renin-angiotensin system blockers. Although an intervention targeting direct reduction in oxidative stress by antioxidants is attractive, clinical studies in which the efficacy of oral administration of antioxidants against diabetic vascular complications was investigated have yielded disappointing results. Further studies are needed to develop therapeutic strategies for improving endothelial function and cardiovascular outcomes by decreasing oxidative stress in patients with diabetes.

## Figures and Tables

**Table 1:** Hypoglycemic drugs and their effects on endothelial function.

Hypoglycemic Drugs	Effects on Endothelial Function
Insulin therapy [44]	Endothelial function is potentially impaired due to the selective insulin resistance in patients with insulin resistance.
Sulfonylureas [44]	Endothelial function is potentially impaired due to the selective insulin resistance in patients with insulin resistance.
Glinides [45]	∙ Endothelial function is potentially improved by reducing glucose fluctuations through decreasing postprandial hyperglycemia. ∙ Endothelial function is potentially impaired due to the selective insulin resistance in patients with insulin resistance.
α-glucosidase inhibitors [45,46,47]	Endothelial function is potentially improved by reducing glucose fluctuations through decreasing postprandial hyperglycemia.
DPP-4 inhibitors [46,48,49,50]	∙ Endothelial function is potentially improved by reducing glucose fluctuations through decreasing postprandial hyperglycemia. ∙ Endothelial function is potentially impaired due to the selective insulin resistance in patients with insulin resistance.
GLP-1R agonists [51,52,53,54,55]	Endothelial function is potentially improved by reducing glucose fluctuations through decreasing postprandial hyperglycemia, by reducing postprandial triglycerides levels, and by activating AMPK.
Thiazolidinediones [56,57]	Endothelial function is potentially improved by reducing insulin resistance.
Metformin [58,59,60,61,62,63,64,65,66]	Endothelial function is potentially improved by reducing insulin resistance, activating AMPK and sirtuin-1, and promoting antioxidation.
SGLT2 inhibitors [67,68,69,70,71,72,73,74,75,76,77,78]	Endothelial function is potentially improved by lowering glucose levels in an insulin-independent manner, reducing acute glucose fluctuations, improving insulin sensitivity, and improving other metabolic parameters.

DPP-4 indicates dipeptidyl peptidase 4; GLP-1R, glucagon-like peptide 1 receptor; SGLT2, sodium-glucose cotransporter 2; AMPK, AMP-activated protein kinase.

## Abbreviations

NO = nitric oxide, ET-1 = endothelin-1, ROS = reactive oxygen species, NADPH = nicotinamide-adenine dinucleotide phosphate, eNOS = endothelial nitric oxide synthase, BH_4_ = tetrahydrobiopterin, TCA = tricarboxylic acid, NADH = nicotinamide adenine dinucleotide, FADH_2_ = 1,5-dihydro-flavin adenine dinucleotide, PKC = protein kinase C, AGE = advanced glycation end products, HbA1c = glycated hemoglobin, IRS = insulin receptor substrates, PI3-kinase = phosphoinositide 3-kinase, MAPK = mitogen-activated protein kinase, ERK = extracellular signal-regulated kinase, DPP-4 = dipeptidyl peptidase 4, GLP-1R = glucagon-like peptide 1 receptor, AMPK = AMP-activated protein kinase, SGLT2 = sodium-glucose cotransporter 2.
